# Osilodrostat Treatment for Adrenal and Ectopic Cushing Syndrome: Integration of Clinical Studies With Case Presentations

**DOI:** 10.1210/jendso/bvaf027

**Published:** 2025-02-14

**Authors:** Maria Fleseriu, Richard J Auchus, Irina Bancos, Beverly M K Biller

**Affiliations:** Pituitary Center, Departments of Medicine and Neurological Surgery, Oregon Health & Science University, Portland, OR 97239, USA; Department of Pharmacology, University of Michigan, Ann Arbor, MI 48109, USA; Division of Metabolism, Endocrinology and Diabetes, University of Michigan, Ann Arbor, MI 48105, USA; Division of Endocrinology, Metabolism and Nutrition, Mayo Clinic, Rochester, MN 55905, USA; Neuroendocrine and Pituitary Tumor Clinical Center, Massachusetts General Hospital, Boston, MA 02114, USA

**Keywords:** ectopic ACTH syndrome, adrenal Cushing syndrome, medical treatment, osilodrostat, clinical practice, case studies

## Abstract

Although most cases of endogenous Cushing syndrome are caused by a pituitary adenoma (Cushing disease), approximately one-third of patients present with ectopic or adrenal causes. Surgery is the first-line treatment for most patients with Cushing syndrome; however, medical therapy is an important management option for those who are not eligible for, refuse, or do not respond to surgery. Clinical experience demonstrating that osilodrostat, an oral 11β-hydroxylase inhibitor, is effective and well tolerated comes predominantly from phase III trials in patients with Cushing disease. Nonetheless, reports of its use in patients with ectopic or adrenal Cushing syndrome are increasing. These data highlight the importance of selecting the most appropriate starting dose and titration frequency while monitoring for adverse events, including those related to hypocortisolism and prolongation of the QT interval, to optimize treatment outcomes. Here we use illustrative case studies to discuss practical considerations for the management of patients with ectopic or adrenal Cushing syndrome and review published data on the use of osilodrostat in these patients. The case studies show that to achieve the goal of reducing cortisol levels in all etiologies of Cushing syndrome, management should be individualized according to each patient’s disease severity, comorbidities, performance status, and response to treatment. This approach to osilodrostat treatment maximizes the benefits of effective cortisol control, leads to improvements in comorbid conditions, and may ameliorate quality of life for patients across all types and severities of Cushing syndrome.

## Background

Endogenous Cushing syndrome (CS) is a rare endocrine disorder caused by hypercortisolism [1-3], which is associated with various comorbidities that negatively affect quality of life (QoL) and survival [1-5]. Mortality risk in CS is 3.5 to 5 times higher than in the general population; the main causes of mortality are cardiovascular disease and infections [1, 6].

**Figure 1.**
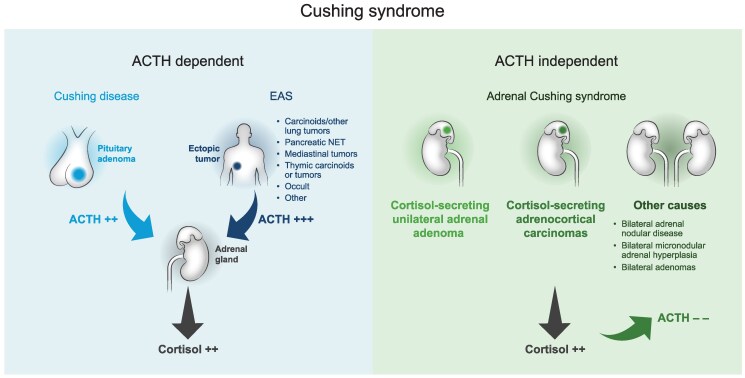
Etiologies of Cushing syndrome [1, 2]. Abbreviations: EAS, ectopic ACTH syndrome; NET, neuroendocrine tumor.

Most cases of endogenous CS are caused by pituitary adenoma (Cushing disease; Fig. 1) [2]; some patients may present with ectopic ACTH syndrome (EAS; ectopic CS) or adrenal tumors [including cortisol-secreting adrenocortical carcinomas (ACCs), unilateral adenomas, and bilateral adrenal nodular disease (BND; also referred to as bilateral macronodular adrenal hyperplasia); Fig. 1] [1, 2]. Incidences of ectopic and adrenal CS relative to Cushing disease have increased recently, which may reflect increased awareness of hypercortisolism in patients with malignant tumors and increased detection of cortisol-secreting adrenal incidentalomas [7, 8]. Compared with other etiologies, patients with EAS or ACCs typically have more severe hypercortisolism, which is associated with higher mortality [1, 9].

**Figure 2.**
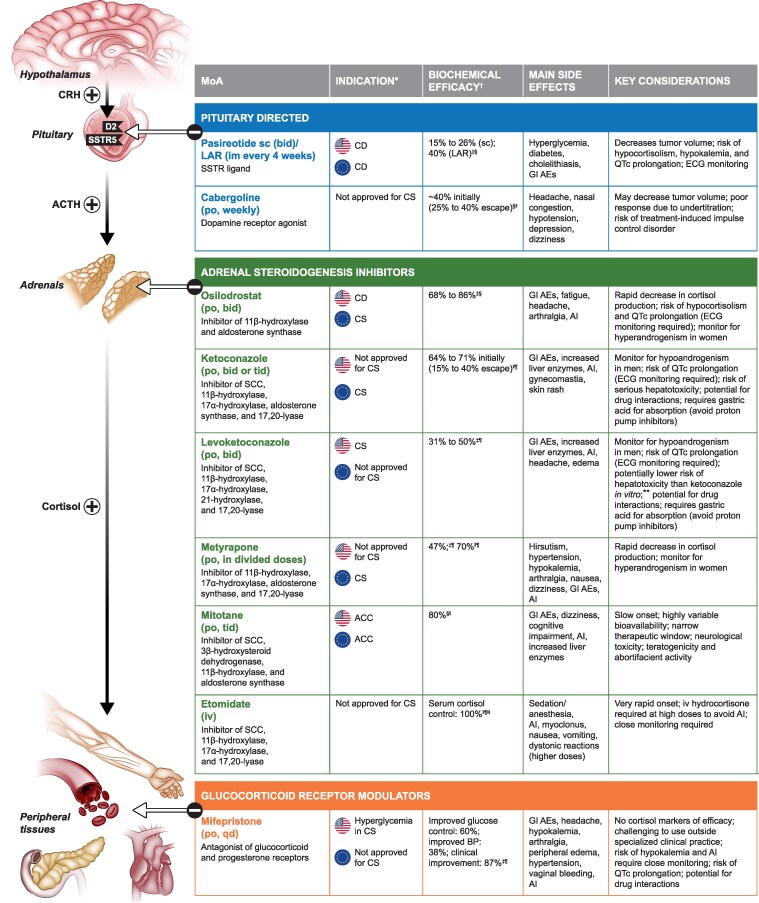
Summary of medical therapies for CS [1, 6, 11-33]. The US prescribing information for levoketoconazole has a black box warning on hepatotoxicity and QT-interval prolongation (the US prescribing information for ketoconazole, indicated for the treatment of fungal infections, also has a black box warning on hepatotoxicity and QT-interval prolongation); the US prescribing information for mifepristone has a black box warning on its antiprogestational effects, which will result in termination of pregnancy. *Specific indications for hypercortisolism are as follows: pasireotide sc/LAR (United States and Europe), adults/patients with CD for whom pituitary surgery is not an option or has not been curative; osilodrostat (United States), adult patients with CD for whom pituitary surgery is not an option or has not been curative; osilodrostat (Europe), adults with endogenous CS; ketoconazole (Europe), endogenous CS in adults and adolescents ≥12 years old; levoketoconazole (United States), endogenous CS in adults for whom surgery is not an option or has not been curative; metyrapone (Europe), endogenous CS; mitotane (United States); inoperable, functional, or nonfunctional ACC; mitotane (Europe), symptomatic treatment of advanced ACC; mifepristone (United States), control of hyperglycemia secondary to hypercortisolism in adults with endogenous CS who have type 2 diabetes mellitus or glucose intolerance, in whom surgery has been noncurative, or who are not candidates for surgery; ^†^proportion of patients with normalization of UFC, unless otherwise specified; ^‡^prospective, phase III study/studies; ^§^in patients with CD; ^∥^retrospective study/studies; ^¶^in patients with CS; **based on animal models (no head-to-head studies in humans available); ^††^serum cortisol level 10 to 20 µg/dL. Abbreviations: ACC, adrenocortical carcinoma; AE, adverse event; AI, adrenal insufficiency; bid, twice daily; BP, blood pressure; CD, Cushing disease; CS, Cushing syndrome; D2, dopamine receptor D2; ECG, electrocardiogram; GI, gastrointestinal; im, intramuscular; LAR, long-acting release; MoA, mechanism of action; po, oral; qd, once daily; QTc, corrected QT; sc, subcutaneous; SCC, side-chain cleavage; SSTR, somatostatin receptor subtype; tid, 3 times daily; UFC, urinary free cortisol.

Normalizing cortisol exposure alleviates comorbidities, improves QoL, and reduces mortality risk [1, 10]. Surgery is the recommended first-line option for most patients with CS [1, 2, 6, 11], but medical therapies (Fig. 2) are important if surgery is not feasible, in cases of postsurgical hypercortisolism recurrence [1, 2, 6, 11, 34-36], and for perioperative management [36]. As there are no randomized head-to-head studies comparing medical therapies for CS, treatment should be individualized, considering the patient’s clinical condition, drug efficacy, rapidity of action, side-effect profile, potential drug interactions, drug availability, and cost [11, 12, 37].

Osilodrostat is a potent oral inhibitor of 11β-hydroxylase, the enzyme that catalyzes the final step of cortisol synthesis [38, 39], and has been evaluated in an extensive clinical trial program of patients with Cushing disease (LINC 1-4) [39-45] and EAS or adrenal CS [46]. These studies demonstrate that osilodrostat provides rapid, sustained cortisol reduction, alongside improvements in cardiovascular and metabolic parameters. Now, real-world experience of osilodrostat in patients with EAS or adrenal CS is emerging (Table 1) [47-62].

**Table 1.** Clinical trial data and real-world experience on the use of osilodrostat in patients with EAS*^a^* or adrenal CS

Study	Patient population and baseline cortisol levels	Duration of osilodrostat exposure and osilodrostat dose	Key efficacy/effectiveness outcomes	Key safety outcomes
EAS*^a^*				
Bessiène et al 2021 [50]	EAS (pancreatic; n = 1) Plasma cortisol: >108.8 µg/dL (>3000 nmol/L), >5.5×ULN	Starting dose 20 mg/day, titrated to 60 mg/day over 5 days, using block-and-replace strategy Follow-up: 20 days	Normalization of cortisol levels within 6 days	Well tolerated (no further information given)
Dormoy et al 2023 [53]	Paraneoplastic CS/EAS with severe hypercortisolism (n = 33) First-line monotherapy (n = 11), second-line monotherapy (n = 13), combination with other medical therapies (n = 9) First-line monotherapy Median (range) UFC: 1678 µg/24 hours (190–32 950) [4631 nmol/24 hours (524–90 942)]; >21.0×ULN Serum cortisol, 35.7 µg/dL (14.1-115.0) [985 nmol/L (389-3172)]; 1.6×ULN Second-line monotherapy 10/13 patients had UFC > ULN before osilodrostat initiation Combination therapy Median (range) UFC 588 µg/24 hours (20–21 448) [1622 nmol/L (55–59 196)]; > 7.4×ULN	First-line monotherapy Median (range) initial dose: 10 mg/day (2-40) Maximum dose: 20 mg/day (10-100) Second-line monotherapy Median (range) initial dose: 4 mg/day (1-20) Maximum dose: 25 mg/day (4-60) Combination therapy Median (range) initial dose: 4 mg/day (1-60) Maximum dose: 40 mg/day (4-80) Dosing strategies employed Osilodrostat titration (n = 6) Block and replace (n = 16) Osilodrostat titration then block and replace (n = 11)	First-line monotherapy Significant reductions in UFC and serum cortisol; UFC normalization in 9/11 patients (81.8%) after median of 2 weeks (range 1-56 weeks) Second-line monotherapy UFC normalized in all 10 patients with levels > UFC at baseline Cortisol control maintained in remaining patients Combination therapy Significant reductions in UFC Overall population Significant reductions in SBP and DBP and decrease in number of antihypertensive medications Significant reductions in FPG and HbA_1c_ and decrease in insulin dose and number of patients who required insulin	Adrenal insufficiency reported in 8/33 patients (24.2%) Strategy for managing hypocortisolism-related events not reported (but authors recommended that patients have access to “stress doses” of glucocorticoid tablets and an emergency injection kit)
Hána et al 2023 [55]	EAS (bronchial neuroendocrine; n = 1) UFC 790 µg/24 hours (2179 nmol/24 hours; 10.5×ULN) and 1230 µg/24 hours (3394 nmol/24 hours; 16.3×ULN)	6 mg/day titrated initially to 45 mg/day within 15 days; tapered down and eventually stopped at day 52; reintroduced at 15 mg/day on day 85 (because of an increase in cortisol); reduced to 5 mg/day 4 weeks later Follow-up: 6 months	Day 12 (osilodrostat dose 45 mg/day): UFC normalized Month 4 (osilodrostat dose 45 mg/day): Morning cortisol and UFC in normal ranges Reduction in blood pressure following alleviation of hypercortisolism Improvements in other clinical signs of hypercortisolism	Patient experienced symptoms of hypocortisolism (nausea and weakness), treated with temporary hydrocortisone replacement
Heleno et al 2023 [56]	EAS (lung adenocarcinoma; n = 1) Cortisol: 50.3 μg/dL (1388 nmol/L; 2.0×ULN) UFC: 2835 μg/24 hours (7821 nmol/24 hours; 56.7×ULN)	2 mg bid Duration of follow-up not reported	After 1 month, hypercortisolism symptoms improved and UFC decreased to 53 μg/24 hours (146 nmol/24 hours)	Not reported
Sawabe et al 2024 [58]	EAS (SCLC; n = 1) Markedly elevated plasma ACTH [770 pg/mL (170 pmol/L); 12.2×ULN] and serum cortisol levels [88.3 µg/dL (2436 nmol/L); 4.5×ULN]	Starting dose 1 mg/day, titrated to 20 mg/day (patient was also taking metyrapone, the dose of which was decreased and then discontinued 20 days after starting osilodrostat) Dose gradually downtitrated (based on serum cortisol levels) to 1 mg/day, 66 days after osilodrostat initiation (no further information available on rate of decrease) Discontinued after day 128	Full suppression of serum cortisol from day 48 [1.5 µg/dL (41.4 nmol/L)] at a dose of 20 mg/day No increase in serum cortisol following dose reduction to 1 mg/day Serum cortisol levels remained below 4.0 μg/dL (110 nmol/L) after osilodrostat discontinuation (reason for discontinuation not specified) Note that the patient was also receiving chemotherapy with etoposide and carboplatin	Not reported
Adrenal CS				
Amodru et al 2021 [47]	BND (n = 1) Morning (08:00) serum cortisol: 23.9 µg/dL (658 nmol/L)*^b^* Midnight serum cortisol: 23.1 µg/dL (637 nmol/L)*^b^* LNSC: 3.1 µg/dL (85 nmol/L)*^b^*	Combination of ketoconazole (600 mg/day) and osilodrostat (30 mg/day) Treatment duration not reported	Morning (08:00) and midnight serum cortisol levels decreased to 15.8 µg/dL (436 nmol/L) and 6.2 µg/dL (172 nmol/L), respectively Rapid improvement in hypertension, hypokalemia, and diabetes control	No AEs
Malik et al 2022 [57]	Adrenal adenoma (n = 1) UFC (at diagnosis): 1324 μg/24 hours (3652 nmol/24 hours; 26.5×ULN) Morning (08:00) cortisol after 1 mg DST (at diagnosis): 13.8 μg/dL (380.7 nmol/L; 7.7×ULN) Morning (08:00) cortisol (treatment day 1): 19.2 μg/dL (529.7 nmol/L)*^b^*	2 mg bid initially, increased to 4 mg bid on day 5, then reduced to 2 mg bid on day 10 Duration of osilodrostat treatment: 22 days	Morning cortisol decreased to 5.1 μg/dL (140.7 nmol/L) on treatment day 22 Decrease in fasting glucose from 224 mg/dL (12.4 mmol/L) to 122 mg/dL (6.8 mmol/L)	Adrenal insufficiency (resolved following osilodrostat dose reduction and temporary hydrocortisone replacement)
Tabarin et al 2022 [60]	ACC (n = 7) Baseline UFC range: 1.8-34.8×ULN*^c^* Baseline cortisol: 35.9 μg/dL (989 nmol/L)*^b^*	Median osilodrostat exposure: 13.7 weeks (range 1-29) Osilodrostat doses ranged from 4 to 40 mg/day	Significant decrease in UFC and/or serum cortisol in 6/7 patients within 2 weeks Maximal decrease in UFC from 10.5 to 0.6×ULN (*P* = 0.03) Decrease in mean serum cortisol to 12.0 µg/dL (330 nmol/L; *P* = 0.02) Time to full control of hypercortisolism: 1 week to 3 months Improvements in clinical appearance, blood pressure, and potassium levels	Mild transient adrenal insufficiency in 3/7 patients, managed with osilodrostat dose reduction or temporary hydrocortisone replacement No discontinuations because of side effects
Stasiak et al 2024 [59]	Adrenal adenoma (n = 2), preoperative management Patient 1 UFC 310 µg/24 hours (856 nmol/24 hours; 1.8×ULN) Morning serum cortisol: 16.0 μg/dL (441.4 nmol/L)*^b^* Late-night (midnight) serum cortisol: 13.5 μg/dL (372.4 nmol/L)*^b^* Patient 2 UFC: 725 µg/24 hours (2000 nmol/24 hours; 4.1×ULN) Morning (08:00) serum cortisol: 17.94 μg/dL (494.9 nmol/L)*^b^* Late-night (midnight) serum cortisol: 16.11 μg/dL (444.4 nmol/L)*^b^*	Patient 1 Initial dose 1 mg bid, gradually increased to 6 mg/day then 8 mg/day, then rapid increase to 45 mg/day following adenoma progression Patient 2 Initial dose 2 mg/day, titrated to 45 mg/day No further information available on time course of dose changes in either patient	Patient 1 Initial increase in cortisol levels because of adenoma progression, but normalized when osilodrostat dose was increased to 45 mg/day, ∼2 months after starting osilodrostat [UFC 168 µg/24 hours (463.4 nmol/24 hours)] Improvements in potassium levels and blood pressure after ∼2 months of osilodrostat treatment Patient 2 UFC normalized 1 week after administration of osilodrostat 45 mg/day	Patient 1 No worsening AEs during dose escalation to 45 mg/day Patient 2 No AEs
Mixed etiologies				
Haissaguerre et al 2020 [54]	Total, n = 3 – EAS (SCLC; n = 2) – ACC (n = 1) Plasma cortisol [normal range: 7.3-18.1 μg/dL (200-500 nmol/L)]: – Patient 1: 43.5 μg/dL (1200 nmol/L; 2.4×ULN) – Patient 2: 54.2 μg/dL (1495 nmol/L; 3.0×ULN) – Patient 3: 71.4 μg/dL (1970 nmol/L; 3.9×ULN)	Patient 1: starting dose 2 mg/day, titrated to 25 mg/day over 2 weeks Patient 2: osilodrostat (starting dose 5 mg/day, titrated to 44 mg/day over 2 weeks) plus mitotane (1500 mg/day), with high-dose hydrocortisone (60 mg/day) added as part of block-and-replace regimen when plasma cortisol started to decrease below normal Patient 3: osilodrostat (starting dose 1 mg/day, titrated to 7 mg/day) plus ketoconazole	Rapid control of severe hypercortisolism in all 3 patients Time to control of plasma cortisol ≤2 weeks	Excellent tolerability, with no reports of nausea, changes in QTc interval, or hypokalemia
Tanaka et al 2020 [46]	Total, n = 9 – Adrenal adenoma (n = 5) – EAS (n = 3) – ACTH-independent macronodular adrenal hyperplasia (n = 1) mUFC: 100.7-3852.9 µg/24 hours (277.9–10 595.6 nmol/24 hours; 2.0-77.1×ULN)	Median (range) average dose: 2.1 mg/day (1.2-7.5) Median (range) osilodrostat exposure: 12 weeks (1.3-81.9)	Median (range) percentage reduction in mUFC at week 12: 94.5% (99.0-52.6) 6/9 patients (66.7%) had mUFC ≤ ULN at week 12	Most frequent AEs were adrenal insufficiency (n = 7), GGT increase, malaise, and nasopharyngitis (n = 3 each) Steroid-withdrawal syndrome was reported in 1 patient Most hypocortisolism-related AEs were managed with osilodrostat dose adjustment or interruption and temporary glucocorticoid replacement; none required treatment discontinuation
Auchus et al 2022 [48] (ILLUSTRATE)	Total, n = 42 – Cushing disease (n = 34) – Adrenal CS (n = 5) – EAS (n = 3) UFC 0.42-27.76×ULN (adrenal, n = 4) and 2.57-75.2×ULN (ectopic)*^bc^*	Starting doses: 1-4 mg/day (adrenal); 2 mg bid (ectopic) Duration of treatment not reported	Adrenal CS (n = 1): UFC maintained at ≤ ULN during osilodrostat treatment in patient who had received prior medical therapy EAS (n = 2): substantial UFC reductions, from 57.1×ULN to 2.9×ULN at day 91 and 75.2×ULN to 0.076×ULN at day 134	Generally well tolerated One case of adrenal insufficiency, managed with treatment interruption Two patients with symptoms suggestive of GWS, 1 requiring treatment interruption
Bonnet-Serrano et al 2022 [51]	Total, n = 33 Osilodrostat group Cushing disease (n = 16) EAS (n = 3) Metyrapone group Cushing disease (n = 7) EAS (n = 1) Metyrapone followed by osilodrostat group Cushing disease (n = 6) Patients in this group were analyzed according to the treatment they received at each follow-up Baseline cortisol levels not reported	Median (range) dose: 10 mg/day (2-40) Median (range) duration: 7 months (0.75-96)	Median UFC: osilodrostat, 14.4 μg/24 hours (39.8 nmol/24 hours); metyrapone, 16.6 μg/24 hours (45.8 nmol/24 hours)	Testosterone levels in women were significantly greater in the metyrapone group Hypocortisolism [cortisol <3.6 μg/dL (<100 nmol/L)] occurred in 48% of osilodrostat-treated patients and 7% of metyrapone-treated patients Strategy for managing hypocortisolism not reported
Detomas et al 2022 [52]	Total, n = 8*^d^* – Cushing disease (n = 5) – EAS (n = 1) – Adrenal adenoma (n = 1) – ACC (n = 1) Mean (SEM) serum cortisol: 22.8 µg/dL (3.5) [629.0 nmol/L (96.6)]; 0.9×ULN Mean (SEM) serum cortisol after 1 mg DST: 12.6 µg/dL (5.7) [347.6 nmol/L (157.2)]*^b^* Mean (SEM) UFC: 817 µg/24 hours (644) [2254 nmol/L (1777)]; 11.7×ULN Mean (SEM) LNSC: 0.9 µg/dL (0.5) [24.8 mmol/L (13.8)]*^b^*	Median (range) doses (mg/day): – Week 2, 4.0 (3.0-7.0) – Week 4, 6.0 (4.0-20.0) – Week 12, 7.0 (6.0-10.0) Block-and-replace strategy used in several patients Duration of treatment: 12 weeks	Mean (SEM) UFC decreased to 131 µg/24 hours (55) [361.4 nmol/L (151.7)] at week 12 2/4 patients (50.0%) had normalized UFC at week 12 Mean (SEM) serum cortisol decreased to 13.0 µg/dL (1.6) [358.6 nmol/L (44.1)] at week 12 Reduction in blood pressure at week 4	Osilodrostat discontinued in 1 patient because of AEs (depression, asthenia, nausea) and in 2 patients after tumor resection Progressive increase in QTc interval over 12 weeks to 432 ms; treatment temporarily interrupted in 1 patient
Bancos et al 2024 [49] (LINC 6)	Total, n = 94 (1-year interim analysis) – Cushing disease (n = 78) – Adrenal adenoma (n = 3) – Adrenal hyperplasia (n = 3) – EAS (n = 9) – Other (n = 1) Baseline cortisol levels not reported	Median (min–max) osilodrostat exposure: 5.5 months (0.1-13.9) Median (min–max) osilodrostat dose: 5.0 mg/day (1.0-60.0)	At month 3, mUFC, serum cortisol, and LNSC were normalized in 71.4% (n = 10/14), 69.2% (n = 18/26), and 50.0% (n = 7/14) of patients, respectively	Most common treatment-related AEs: vomiting and dizziness (both n = 4/44; 9.1%) AEs related to hypocortisolism, accumulation of adrenal hormone precursors, QT prolongation, and pituitary tumor enlargement occurred in 7 (7.4%), 4 (4.3%), 2 (2.1%), and 0 patients, respectively
Tabarin et al 2024 [61] (LINC 7)	Total, n = 103 – EAS (n = 53) – ACC (n = 19) – Adrenal adenoma (n = 17) – BND (n = 14) Mean (SD) mUFC: 1518.6 µg/24 hours (3679.8)*^b^* [4189.8 nmol/24 hours (10 152.6)]	Median (min–max) osilodrostat dose: 5.0 mg/day (1-60) Median (min–max) osilodrostat exposure: 164 days (1-1178)	Week 12: 23/52 patients (44.2%) had mUFC ≤ ULN Last on-treatment assessment: 33/58 patients (56.9%) had mUFC ≤ ULN and 52/75 (69.3%) had any cortisol measure ≤ ULN	Most common (≥15%) treatment-emergent AEs were adrenal insufficiency (28.2%) and hypokalemia (17.5%) Strategy for managing adrenal insufficiency not reported

Osilodrostat was used off label in some of these cases based on clinical judgment, the patient’s condition, and understanding of osilodrostat pharmacology.

Abbreviations: ACC, adrenocortical carcinoma; AE, adverse event; bid, twice daily; BND, bilateral adrenal nodular disease; CS, Cushing syndrome; DBP, diastolic blood pressure; DST, dexamethasone suppression test; EAS, ectopic ACTH syndrome; FPG, fasting plasma glucose; GGT, gamma-glutamyl transferase; GWS, glucocorticoid-withdrawal syndrome; HbA_1c_, glycated hemoglobin; LNSC, late-night salivary cortisol; mUFC, mean urinary free cortisol; QTc, corrected QT; SBP, systolic blood pressure; SCLC, small-cell lung carcinoma; UFC, urinary free cortisol; ULN, upper limit of normal.

^*a*^The standard term “ectopic ACTH syndrome” has been used to cover the terms used to define the condition in the studies reported (CS from ectopic ACTH secretion; ectopic CS; ectopic corticotropin syndrome).

^*b*^Normal range not reported.

^*c*^Values in µg/24 hours not provided.

^*d*^Study also included 8 patients treated with metyrapone.

As with other reviews describing practical considerations for the use of mifepristone [63], levoketoconazole [13], and pasireotide [64, 65], this narrative review addresses clinical considerations and recommendations for using osilodrostat in patients with EAS or adrenal CS through illustrative patient case studies. These cases have been developed for teaching purposes, based on published case studies and the authors’ clinical experience.

## Case 1: BND

A 60-year-old White female presented with headaches, insomnia, and fatigue. On examination, violaceous striae on flanks and dermal atrophy were noted. Other relevant history included hypertension and depression. Laboratory results were as follows: urinary free cortisol (UFC) 2.3 and 2.9× upper limit of normal (ULN); low ACTH [<2 pg/mL (<0.44 pmol/L)]; serum cortisol 8 μg/dL (220.7 nmol/L) following 1 mg dexamethasone suppression test [DST; normal <1.8 μg/dL (<49.7 nmol/L); dexamethasone level 400 ng/dL (9.0 nmol/L); value for accurate result 100->220 ng/dL (2.25->5.6 nmol/L)] [66, 67]. Blood pressure (BP) was 145/100 mmHg despite ramipril treatment.

Abdominal computed tomography (CT) showed multiple masses on the right (4.2 and 3.1 cm nodules) and left (1.1 and 0.8 cm nodules) adrenal glands. The patient was diagnosed with BND and underwent unilateral laparoscopic adrenalectomy of the right adrenal gland. Glucocorticoid-replacement therapy was administered for 1 year to manage postoperative adrenal insufficiency (AI); following hypothalamus–pituitary–adrenal (HPA) axis recovery, glucocorticoid therapy was stopped.

After 2.5 years, she reported recurrence of mild hypercortisolism symptoms, including a 6 lb weight gain in 2 months, new violaceous striae, and insomnia. UFC was 1.8×ULN, and post-DST cortisol was 2.8 µg/dL (77.3 nmol/L) with appropriate dexamethasone level. CT showed growth of left adrenal nodules to 3.1 and 2.6 cm. She preferred to start medical therapy rather than undergo another adrenalectomy.

Levoketoconazole 150 mg twice daily (bid) was initiated, with some symptom improvement but without UFC normalization. The dose was up-titrated to 300 mg bid, but liver enzymes increased (>3×ULN); treatment was discontinued after 3 months. She switched to metyrapone 250 mg 4 times a day (qid), subsequently increased to 500 mg qid. UFC normalized, but she developed lightheadedness and gastrointestinal disturbances and discontinued treatment after 4 weeks. She switched to osilodrostat following documentation of normal baseline electrocardiogram (ECG) and potassium and magnesium levels. She was educated on hypocortisolism symptoms and given an emergency glucocorticoid kit containing hydrocortisone tablets (20 mg) and hydrocortisone solution for intramuscular injection (100 mg/2 mL). Osilodrostat was initiated at 1 mg bid and titrated to 2 then 4 mg bid over 12 weeks based on UFC and morning serum cortisol levels At 4 mg bid, she experienced progressive fatigue, nausea, anorexia, and dizziness; BP was 90/50 mmHg at home. After consulting her physician, she interrupted osilodrostat treatment and began hydrocortisone 20 mg bid (emergency kit). The following day, morning serum cortisol was 5 µg/dL [138.9 nmol/dL; ULN 20 µg/dL (551 nmol/L)], indicating AI. She continued hydrocortisone 20 mg bid for 3 days. Serum cortisol was normal on day 5, and osilodrostat was restarted at 3 mg bid.

After 12 months, she continued to tolerate osilodrostat 3 mg bid well, with UFC < ULN (0.7 and 0.6×ULN) and no further symptoms of AI. She lost weight, and her sleep improved. BP was controlled, leading to reduction then discontinuation of antihypertensive medication. Corrected QT (QTc) interval and potassium levels remained normal.

### Clinical Case Points

#### Management of patients with BND

The approach to managing BND is more challenging than for unilateral masses [68]. Bilateral adrenalectomy is curative but causes AI requiring lifelong glucocorticoid and mineralocorticoid replacement [1, 69]. Unilateral adrenalectomy is an option [1, 69], particularly in patients with asymmetric disease [70], as in this case. This debulking procedure can induce remission but carries a risk of disease progression [71], as illustrated previously. If progression occurs, ketoconazole, levoketoconazole, or metyrapone can effectively lower cortisol, but certain adverse events (AEs) can occur. In a retrospective study of ketoconazole in Cushing disease, mild and major increases in liver enzymes occurred in 13.5% and 2.5% of patients, respectively [72]. In phase III studies in patients with CS, the most common AEs leading to levoketoconazole discontinuation were liver related [14, 73]. In a phase III/IV study of metyrapone in patients with CS, the most common AEs were nausea (24%) and decreased appetite (18%) [74], and in a retrospective study, the main reasons for metyrapone discontinuation were gastrointestinal upset and dizziness [75].

#### Osilodrostat dose-titration regimen

In the United States, the recommended starting dose is 2 mg bid, titrated by 1 to 2 mg bid no more frequently than every 2 weeks. In Europe, the recommended starting dose is 2 mg bid, with dose titration by 1 or 2 mg according to response (monitored every 1-2 weeks until adequate clinical response maintained). Dose titration should be individualized and guided by several cortisol assessments [eg, 24-hour UFC, late-night salivary cortisol (LNSC), and serum/plasma cortisol] and clinical response. For Asian patients, a lower starting dose (1 mg bid) is recommended because of greater sensitivity to osilodrostat vs non-Asian patients [76]. In this White patient, osilodrostat was started at 1 mg bid, because tumor progression was diagnosed early when hypercortisolemia was mild, and titrated over 12 weeks to 4 mg bid.

#### AI with steroidogenesis inhibitors

AI is an anticipated side effect that can occur when the dose of steroidogenesis inhibitors is higher than needed or if patients have intercurrent illness [1, 2, 6, 77]. Symptoms include fatigue; appetite loss; gastrointestinal symptoms; and abdominal, muscle, and joint pain [77]. Serum cortisol levels that are low or near the lower limit of normal [eg, <5-10μg/dL (<140-280 nmol/L); normal range 5-25 μg/dL (140-690 nmol/L)] are required to confirm AI; however, presumptive treatment takes priority over laboratory testing when patients have vomiting, hypotension, or hypoglycemia. Patients should be educated on the symptoms and situations that can precipitate AI (eg, infection, surgery, injury, hemorrhagic shock) and prescribed an emergency home glucocorticoid kit to allow timely glucocorticoid initiation [78-80].

### Published Osilodrostat Data

#### Use in patients with BND

Osilodrostat data in patients with BND are limited. In a phase II study, 1 patient with BND sustained normalization of serum cortisol over 1.5 years (osilodrostat doses, 0.5-6 mg/day) [46]. In a case report of a patient with BND treated with combined osilodrostat (30 mg/day) and ketoconazole (600 mg/day), morning and midnight serum cortisol decreased during treatment [47]. Rapid improvements in hypertension, hypokalemia, diabetes, and lower-extremity edema were observed; no side effects were reported [47]. In the retrospective LINC 7 study, 14/103 patients had BND, but outcome data from this subgroup are not yet available [61].

#### AI

In LINC 3 and 4, 54.0% and 27.4% of patients with Cushing disease, respectively, reported hypocortisolism-related AEs, mainly during initial dose titration. Events were generally mild or moderate; 4% of patients discontinued for this reason [42-45]. These events were investigator reported, with no protocol-mandated requirement to diagnose AI based on serum cortisol [42, 43, 45]. The lower incidence in LINC 4 may reflect the slower dose-escalation schedule (every 3 vs 2 weeks in LINC 3) [43-45]. There have been a few reports of prolonged hypocortisolism for ≤15 months after osilodrostat discontinuation [81, 82]; further research to elucidate the underlying mechanisms is needed.

#### Improvements in BP

Hypertension is a common comorbidity in CS, affecting 58% to 85% of patients at diagnosis [4]. Many steroidogenesis inhibitors can lead to accumulation of mineralocorticoid precursors, potentially increasing BP. However, this is counterbalanced by BP reduction from cortisol-lowering effects [83].

Overall, BP decreased during the LINC 3 and 4 core phases; reductions were sustained during long-term treatment [42-45]. In a pooled analysis of LINC 3 and 4, BP decreased to below hypertensive values in ≥50% of patients with high baseline BP. Some patients reduced or stopped antihypertensive medication [84].

## Case 2: EAS Caused by Lung Neuroendocrine Tumor (NET)

A 66-year-old male presented with rapidly progressing, severe proximal myopathy, weight loss, capillary fragility, asthenia, insomnia, and infections over 6 weeks and a recent vertebral fracture. He was a smoker and had diabetes treated with metformin, sitagliptin, and insulin. Laboratory results were as follows: morning serum cortisol 65 µg/dL [1793 nmol/L; ULN 20 µg/dL (551 nmol/L)]; UFC 18.1×ULN; LNSC 460 ng/dL [12 690 nmol/L; ULN 100 ng/dL (2759 nmol/L)]; ACTH 210 pg/mL [46.2 pmol/L; ULN 55 pg/mL (21.1 pmol/L)]; fasting plasma glucose (FPG) 211 mg/dL (11.7 mmol/L); glycated hemoglobin (HbA_1c_) 9.5%; severe hypokalemia [2.1 mmol/L (2.1 mEq/L); normal range, 3.7-5.2 mmol/L (3.7-5.2 mEq/L)] [85]. Following thoracic magnetic resonance imaging, ^68^Ga-DOTATATE positron emission tomography (PET)-CT, and biopsy, he was diagnosed with small-cell lung carcinoma (SCLC).

He was admitted to the hospital and started on chemotherapy (carboplatin/etoposide) as he was too frail for lung surgery or bilateral adrenalectomy. He was given high doses of potassium (120 mEq/day) and spironolactone (100 mg/day) for hypokalemia, anticoagulants for high thromboembolic risk, and sulfamethoxazole-trimethoprim (Bactrim) for prevention of *Pneumocystis jirovecii* pneumonia.

Following evidence of normal baseline ECG and potassium and magnesium levels, he started osilodrostat 5 mg bid and concomitant hydrocortisone (20 mg bid as part of a block-and-replace regimen) while initiating chemotherapy. Given the concomitant start of chemotherapy, which has potentially severe AEs, and the patient’s history of recent infections, a slightly supraphysiologic hydrocortisone dose to prevent adrenal crisis was selected at the beginning of the treatment. Osilodrostat was titrated to 10 mg bid over 4 days, with daily monitoring of morning serum cortisol. He was switched from hydrocortisone 20 mg bid to dexamethasone 0.5 mg/day to avoid cross-reaction with the cortisol assay while uptitrating the osilodrostat dose. When he developed a fever, dexamethasone was temporarily further increased to 0.75 mg/day for several days and then, upon fever recovery, was switched back to hydrocortisone 20 mg bid, with the intention of decreasing the hydrocortisone dose to 20 mg/day when overall clinical status improved and supraphysiologic glucocorticoid doses were no longer needed.

During osilodrostat titration, morning serum cortisol normalized. He experienced QTc prolongation; given his high cortisol levels and the ability to closely monitor the QTc interval during inpatient admission, osilodrostat was continued but at a reduced dose (8 mg bid). QTc prolongation resolved following dose reduction, and he continued osilodrostat 8 mg bid. Nine months after osilodrostat initiation, serum cortisol and UFC were normal. HbA_1c_ decreased to 7.4%, leading to insulin discontinuation; metformin-sitagliptin treatment was maintained. Potassium levels normalized; potassium supplementation was decreased to 40 mEq/day and spironolactone gradually downtitrated to 25 mg/day. He experienced improvements in sleep and muscle strength, permitting some mobility and improved well-being. However, chemotherapy failed, and he died 10 months later from SCLC progression.

### Clinical Case Points

#### Cortisol-lowering therapy as first-line CS treatment

In patients with EAS and localized tumor, surgery is recommended to remove primary lesions [1]. However, surgery may not be curative when there are known metastases, and in ≤20% of ectopic cases, the tumor cannot be located [86]; in these circumstances, bilateral adrenalectomy may be considered [53, 87, 88]. Alternatively, first-line cortisol-lowering therapy can be used [89], especially for patients with severe disease and/or at high surgical risk.

In patients who require rapid cortisol normalization or blockade, treatment options include oral steroidogenesis inhibitors or mifepristone (if hyperglycemia is also present) and, for inpatients, the intravenous steroidogenesis inhibitor etomidate [11, 78, 90-92]. Combination therapy (ketoconazole and metyrapone [93]; mitotane, ketoconazole, and metyrapone [94]) has also been used to rapidly control cortisol in patients with severe hypercortisolism from EAS.

#### Block and replace

For patients who need urgent cortisol reduction, higher starting doses and faster titration of cortisol-lowering therapy may be required, as in this case. This may increase the AE risk, including AI [78], QTc prolongation, and hypokalemia. To mitigate the AI risk, glucocorticoid coadministration with cortisol-lowering therapy (block-and-replace strategy) has been described in the literature [53, 78], as has titration followed by glucocorticoid replacement [53].

A proposed algorithm for osilodrostat in patients with EAS recommends hydrocortisone (20-30 mg/day), prednisone (4-7 mg/day), or dexamethasone (0.5 mg/day) as part of the block-and-replace strategy [53], although doses may vary. Hydrocortisone and prednisone interfere with serum cortisol immunoassays [53], an important consideration particularly early on in steroidogenesis inhibitor uptitration with a block-and-replace regimen, when serum cortisol measurement is essential. However, their short half-lives allow the timing of the blood draw to early-morning sampling, > 24 hours after the last dose, to avoid interference. Patients treated with block and replace should be advised that replacement glucocorticoid doses may need to be increased during periods of stress or acute illness [53, 78].

#### Measurement of cortisol

CS diagnosis can be based on UFC, LNSC, and/or DST [11]. For UFC and LNSC, intraindividual variability is high, so the average of 2 to 3 samples is recommended [11, 78, 95], but in the severe case described here, single tests were sufficient. UFC is used to monitor treatment response; LNSC can also be informative and does not always reflect changes in UFC [78]. In a pooled analysis of LINC 3 and 4, patients with both mUFC and LNSC controlled had greater improvements in cardiovascular/metabolic-related parameters than those with only mUFC controlled or both LNSC and mUFC uncontrolled [96]. Measurement of morning serum cortisol is useful if AI is a concern [11, 95]. When monitoring for recurrence, UFC and LNSC elevations may not always be concordant, with some patients showing increased LNSC up to a year before UFC increases [11, 97].

### Published Osilodrostat Data

#### EAS

Data from >100 patients with EAS treated with osilodrostat have been reported in the literature. The results show that osilodrostat effectively lowers cortisol in these patients (Table 1) [46, 48-56, 58, 61]. The results also highlight baseline hypercortisolism variability and different osilodrostat dose regimens, including block and replace to mitigate the risk of AI. However, published safety data in this population are limited.

#### Improvements in glycemic control

Diabetes occurs in 18% to 64% of patients with CS [4]. In LINC 3 and 4, 21.9% and 28.8% of patients, respectively, had diabetes [42, 44]. Early improvements in FPG and HbA_1c_ were sustained during long-term treatment [42-45]. In a pooled analysis of both studies, one-third of patients with high baseline FPG had reductions to normal levels during treatment; some patients reduced or stopped antihyperglycemic medication [84].

#### Improvements in hypokalemia

Hypokalemia is common in patients with hypercortisolism and more prevalent in EAS than in other CS subtypes [98, 99]. Cortisol-lowering treatments can improve hypokalemia, but some may lead to the accumulation of mineralocorticoid precursors, which can lower potassium. Consequently, their overall effect depends on the balance between reduced cortisol levels and mineralocorticoid effects.

In a retrospective study of 33 patients with severe EAS [53], 30 (90.9%) were hypokalemic at diagnosis. Following potassium supplementation or spironolactone, 13 (39.4%) remained hypokalemic [median potassium, 3.8 mmol/L (3.8 mEq/L)]. During osilodrostat treatment, median potassium levels increased to 4.2 mmol/L (4.2 mEq/L), the proportion of patients with hypokalemia reduced to 12.1%, and doses of hypokalemia treatments were decreased. In LINC 3 and 4, potassium levels generally remained stable, within the normal range [43-45]. During the LINC 3 core phase, hypokalemia was reported in 13% of patients and managed with potassium supplements, spironolactone, osilodrostat dose reduction or temporary interruption, or a combination of these approaches [45]. These data highlight the importance of monitoring potassium during osilodrostat treatment [15].

#### Management of QTc prolongation

QTc prolongation is an expected side effect of steroidogenesis inhibitors and requires close monitoring [11]. QTc-prolongation risk is greater when other drugs known to increase the QTc interval are coadministered [12], for example, sulfamethoxazole-trimethoprim [100], as in this case.

In LINC 3 and 4, AEs related to arrhythmogenic potential and QTc prolongation were reported in 6 (4.4%) and 3 patients (4.1%), respectively [43, 45]. In LINC 3, the osilodrostat dose was adjusted or interrupted in 3 patients, and treatment was discontinued in 1 patient [45]. In LINC 4, all cases resolved without treatment discontinuation [43]. These AEs remained infrequent during long-term treatment [42, 44].

In a retrospective analysis of 8 patients with CS treated with osilodrostat, there was a progressive increase in mean QTc interval to 455 ms (SEM 23) after 12 weeks [52]. Treatment was temporarily interrupted in 1 patient, but no patients discontinued [52].

## Case 3: EAS Caused by Pancreatic NET

A 39-year-old female presented with a history of unexplained weight gain, facial plethora, dorsocervical and supraclavicular fat pads, hair thinning, bruising, and dyspepsia. She also reported forgetfulness, lack of concentration, feeling “spaced out,” and poor QoL. She had new-onset hypertension, type 2 diabetes, and osteoporosis with previous atraumatic femoral fracture. She had an intrauterine device and was not taking oral contraceptives. Laboratory results were as follows: morning serum cortisol 30 μg/dL [827 nmol/L; ULN 23 µg/dL (634 nmol/L)]; ACTH 90 pg/mL [19.8 pmol/L; ULN 52 pg/mL (11.4 pmol/L)]; UFC 3.2×ULN; LNSC 270 ng/dL [74.4 nmol/L; ULN 100 ng/dL (27.6 nmol/L)]; BP 152/99 mmHg; FPG 132 mg/dL (7.3 mmol/L); HbA_1c_ 7.3%.

Pituitary magnetic resonance imaging was negative; bilateral inferior petrosal sinus sampling showed no central gradient. Following ^68^Ga-DOTATATE PET-CT and biopsy, low-grade pancreatic NET with liver metastases was diagnosed. After partial tumor resection and ablation of some metastases, she initiated octreotide depot for the pancreatic NET, atenolol for hypertension, sitagliptin for diabetes, and anticoagulants for high thromboembolic risk.

Metyrapone 250 mg qid was started and well tolerated, with uptitration to 500 mg 3 times a day. UFC normalized, but after 6 months, she complained of hirsutism; testosterone was also elevated. She also reported gastrointestinal upset; metyrapone was therefore discontinued. Ketoconazole and levoketoconazole were considered but discounted because of hepatic dysfunction resulting from metastases. She switched to osilodrostat 2 mg bid (following confirmation of normal baseline ECG and potassium and magnesium levels). The dose was increased every 2 weeks to 4, 6, 8, and then 10 mg bid, based on UFC, until levels normalized. Three weeks after reaching this dose, she experienced fatigue, myalgia, low mood, and severe anorexia; she did not have orthostatic hypotension or low morning serum cortisol [20 µg/dL [552 nmol/L]; 1.2×ULN (ULN 23 µg/dL [634 nmol/L])] and was diagnosed with glucocorticoid-withdrawal syndrome (GWS). Osilodrostat was reduced to 4 mg bid, and GWS symptoms improved. As GWS symptoms abated, the osilodrostat dose was increased to 6 mg bid, with continued titration over 6 months to reach 10 mg bid. After 15 months, the dose was reduced to 8 mg bid; UFC, LNSC, and morning serum cortisol remained in the normal range. She lost weight, and glycemic parameters and BP improved, allowing antihypertensive and antihyperglycemic medication to be reduced and then discontinued. Testosterone decreased to normal levels after 9 months of treatment, and hirsutism improved. She also experienced improvements in memory, concentration, and QoL.

### Clinical Case Points

#### Diagnosis of pancreatic NETs

Somatostatin-receptor PET is preferred for diagnosing most NET types [101]. If unavailable, radiolabeled octreotide scintigraphy or cross-sectional imaging can be used, but sensitivity is lower than with somatostatin-receptor PET [101].

#### Monitoring and managing patients according to aggressiveness of ectopic tumor/hypercortisolism severity

The course of EAS varies by type and tumor grade [102]; low-grade tumors present with a more gradual development of hypercortisolism, while high-grade tumors (eg, SCLC) are associated with severe hypercortisolism and rapid progression [88, 103]. As such, management strategies differ, as illustrated by cases 2 and 3. Patient 3 (outpatient) had milder disease, less frequent monitoring, a lower osilodrostat starting dose, and slower titration.

#### GWS

GWS can occur following surgery or medical treatment for hypercortisolism [104, 105]. GWS and AI can be challenging to differentiate as symptoms overlap, but symptoms such as hypotension, hypoglycemia, and vomiting suggest AI [104, 106, 107]. Furthermore, serum cortisol levels are within or above the normal range with normal glucocorticoid exposure in GWS [104]. Understanding the overlap and differences in symptoms enhances diagnosis and management.

### Published Osilodrostat Data

#### Effect on androgen levels in females

Classical androgen elevations (eg, testosterone) contribute to, but do not fully explain, the clinical symptoms of hyperandrogenism in females with CS. Adrenal 11-oxygenated steroids are biogenic androgens [108] known to drive the hyperandrogenic phenotype in CS [109, 110].

11β-hydroxylase inhibitors, including osilodrostat and metyrapone, can increase levels of androgens and adrenal hormone precursors, analogous to genetic 11β-hydroxylase deficiency. In LINC 3 and 4, testosterone initially increased in females but decreased toward baseline during long-term treatment [42-45]. In the core phases, hirsutism was reported in 11.3% (LINC 3) and 9.6% (LINC 4) of patients; most cases were mild or moderate, and none led to discontinuation [43, 45]. During the extension phases, there was 1 new case of hirsutism in LINC 4 and none in LINC 3 [42, 44]. Furthermore, hirsutism improved during long-term osilodrostat treatment in some female patients with preexisting hirsutism at baseline [42, 44, 111]. In a meta-analysis of LINC 3 and 4 comparing the osilodrostat treatment period (48 weeks) and placebo treatment period (12 weeks), the likelihood of hyperandrogenism was similar between groups [112].

In a retrospective study comparing osilodrostat [median (range) daily dose 10 mg (2-40)] and metyrapone [1250 mg (500-4000)] in 19 patients with ACTH-dependent CS, increases in 11-deoxycortisol and testosterone in female patients were significantly greater with metyrapone than with osilodrostat [51], possibly reflecting longer exposure to metyrapone (33.5 vs 7 months for osilodrostat), differences in their effects on other steroidogenic enzymes (greater, although negligible, inhibition of CYP21A2 and CYP17A1 by osilodrostat), or weak CYP11A1 inhibition by osilodrostat [51, 113]. These data suggest that osilodrostat may be an appropriate alternative for female patients with hyperandrogenism during metyrapone treatment, but further data are needed [51].

#### GWS

Investigator-reported hypocortisolism-related AEs in LINC 3 and 4 [41, 42, 44, 45], including GWS, were summarized in case 1. Patients must be monitored for symptoms of GWS and AI, with regular assessment of serum cortisol levels if symptoms occur, to help differentiate between GWS (normal or high) and AI (below or near lower limit of normal).

#### Improvements in QoL

In this case, the patient’s QoL improved during long-term osilodrostat treatment, consistent with results from LINC 3 and 4, in which improvements in Cushing’s Quality-of-Life Questionnaire and Beck Depression Inventory scores were maintained during long-term treatment [42-45, 111].

## Case 4: ACC

A 25-year-old female with a history of weight gain, amenorrhea, hirsutism, and fatigue presented with facial and dorsocervical fat deposits, diffuse striae, acne, bruising, hirsutism, and proximal muscle weakness. Laboratory results were as follows: UFC 6.9×ULN; morning serum cortisol 52 μg/dL [1434 mmol/L; ULN 24 μg/dL (662 mmol/L)]; ACTH <2 pg/mL [<0.44 pmol/L; normal range 9-52 pg/mL (2.0-11.5 pmol/L)]; potassium 2.9 mmol/L [2.9 mEq/L; normal range, 3.7-5.2 mmol/L (3.7-5.2 mEq/L)] [85] despite potassium supplementation (80 mEq bid). CT showed a 7 cm heterogeneous left adrenal mass with punctate lung metastases, and stage IV ACC was diagnosed.

She started mitotane in combination with osilodrostat (after obtaining baseline ECG and potassium and magnesium levels, which were normal). Osilodrostat was initiated at 4 mg bid, uptitrated by 2 mg bid every 2 to 4 weeks, based on UFC levels. Modest reductions in UFC and serum cortisol were observed at week 2, with further reductions following subsequent dose increases; UFC normalized by month 4 with osilodrostat 12 mg bid. After 10 months, the cancer progressed, with liver and bone metastases; cisplatin, doxorubicin, and etoposide were added. Two months later (12 months after osilodrostat initiation), ACTH rose to 16 pg/mL (3.52 mmol/L); UFC, morning serum cortisol, and LNSC were normal. Potassium levels were within normal range, and potassium supplementation was reduced. Signs and symptoms, including myopathy and bruising, also improved.

### Clinical Case Points

#### Mitotane in ACC management

Mitotane, a steroidogenesis inhibitor, has a long-lasting adrenolytic action on steroid-secreting adrenocortical cells [11], leading to ACC tumor regression in 25% of cases and cortisol reduction in most patients [114]. However, it has highly variable bioavailability and slow onset [16]. In this case, osilodrostat was given concomitantly to reduce cortisol levels rapidly while awaiting the effect of mitotane. Mitotane is a potent inducer of CYP3A4, which can markedly increase the clearance of other drugs metabolized by this isoenzyme [16, 115], including osilodrostat, explaining the higher-than-recommended starting dose (Table 1). If tumor progression occurs during mitotane treatment, chemotherapy with etoposide, doxorubicin, and cisplatin can be added [116].

### Published Osilodrostat Data

#### Patients with ACC

The efficacy and tolerability of osilodrostat in patients with ACC was evaluated in 7 patients with baseline UFC of 1.8 to 34.8×ULN [60]. All patients achieved cortisol control with osilodrostat 4 to 40 mg/day; in 6 patients, UFC and/or serum cortisol significantly decreased after 2 weeks of treatment. Improvements in symptoms, BP, glycemic control, and hypokalemia were observed. Mild transient AI occurred in 3 patients, and worsening hypokalemia developed in 1 patient who also received cabozantinib. No patients discontinued because of AEs [60].

## Case 5: Adrenal Incidentaloma

A 50-year-old female presented with abdominal pain. Medical history included central adiposity (body mass index 51 kg/m^2^), dyslipidemia, hepatic steatosis, hypertension, obstructive sleep apnea, and poorly controlled diabetes. Abdominal CT identified a 2.6 cm left adrenal mass. Laboratory results were as follows: morning serum cortisol and UFC normal; early-morning ACTH 9 to 12 pg/mL [1.98-2.64 pmol/L; normal range 10-50 pg/mL (2.2-11.0 pmol/L)]; dehydroepiandrosterone sulfate (DHEAS) 24 to 30 μg/dL [0.65-0.81 μmol/L; normal range for females aged 50-59 years 15-170 µg/dL (0.41-4.59 μmol/L)]; DST cortisol 2.1 to 2.4 μg/dL (58.0-66.2 nmol/L) with appropriate dexamethasone level [normal <1.8 μg/dL (<49.7 nmol/L)]; FPG 131 mg/dL (7.3 mmol/L); HbA_1c_ 8.5%; BP 150/95 mmHg.

She was diagnosed with functioning benign adrenal incidentaloma with mild autonomous cortisol secretion (MACS). Given her high body mass index, the physician outlined the risks of surgery, and she chose medical management. Mifepristone was initiated, and diabetes improved; however, she discontinued treatment because of vaginal bleeding. She switched to osilodrostat following documentation of normal baseline ECG and potassium and magnesium levels. She was educated on hypocortisolism symptoms and given a home emergency glucocorticoid kit. Osilodrostat was initiated at 1 mg every other day, uptitrated by 1 mg every 2 weeks to 2 mg bid, based on morning serum cortisol levels. One week later, she experienced fatigue, nausea, and low BP at home. On her physician’s advice, she started hydrocortisone 20 mg/day in the morning. The following day, morning serum cortisol was 7 μg/dL (193 mmol/L), indicating possible AI. Osilodrostat was interrupted. Two weeks later, when cortisol levels normalized and symptoms resolved, hydrocortisone was stopped and osilodrostat restarted at 1 mg every other day (bedtime). Morning serum cortisol was monitored regularly, and osilodrostat was increased to 1 mg/day at bedtime 4 weeks later.

Twelve months after switching to osilodrostat, predose morning serum cortisol remained normal; ACTH and DHEAS increased to mid-normal ranges. She experienced weight loss and improvements in glycemic parameters and BP; consequently, the antihyperglycemic medication dose was reduced, and antihypertensive treatment was stopped.

### Clinical Case Points

#### Medical vs surgical management of MACS

The European Society of Endocrinology guidelines on managing adrenal incidentalomas with confirmed MACS suggest considering adrenalectomy in patients with MACS-associated comorbidities, depending on individual patient factors [117]. Circadian cortisol rhythm is abnormal in these patients [118] and has been shown to normalize following 2 evening doses of metyrapone [119]. The role of cortisol-directed therapy vs treatment of comorbidities is controversial, however, and requires further study.

In patients with normal UFC but an abnormal HPA axis, there is a risk of AI following surgery or cortisol-lowering therapy. This risk may be greater in those with higher cortisol levels post-DST and lower plasma ACTH (or serum DHEAS) levels preoperatively [117], but the need for cortisol replacement after adrenalectomy is difficult to predict [120]. Guidelines recommend glucocorticoid replacement during and after adrenalectomy, with postoperative doses tapered by an experienced physician only after documented HPA axis recovery [117].

#### Mifepristone for MACS

Mifepristone binds to glucocorticoid receptors and blocks cortisol action rather than lowering cortisol, which is the mechanism of other treatments for CS [63].

Mifepristone was the first-line treatment in this case, based on its beneficial effects on comorbidities [63, 92, 121]. In a small pilot study of 8 patients with MACS, significant reductions in FPG and insulin resistance were observed after 3 to 6 months of mifepristone [122].

### Published Osilodrostat Data

Published data on osilodrostat in patients with benign solitary adenomas are limited [46, 49, 52, 57, 59, 61]. In this case, osilodrostat was used based on data from studies in Cushing disease, which included some patients with mild hypercortisolism, and on its beneficial effects on comorbidities [42-45, 84, 111]. The patient started on a low dose that was uptitrated every 2 weeks. AI may have been mitigated by slower titration, as shown in LINC 4 vs 3 [43, 45]. Based on the authors’ experience, nighttime administration of osilodrostat is effective and well tolerated in patients with mild adrenal CS.

## Conclusions

These cases of patients with EAS or adrenal CS illustrate that, although reducing cortisol levels is a common goal, management can be complex, and decisions should be individualized to each patient’s condition. Although most available osilodrostat data are in patients with Cushing disease, results from a phase II study, LINC 6, LINC 7, and ILLUSTRATE indicate that osilodrostat is effective and well tolerated in patients with EAS or adrenal CS. Evidence for the use of cortisol-lowering therapies and glucocorticoid-receptor antagonists for MACS is sparse, and further studies are needed to evaluate their role.

Here we have illustrated important considerations for the practical use of osilodrostat, including selection of an appropriate starting dose and titration frequency and monitoring for AEs such as GWS, AI, and QTc prolongation. Such individualized management will maximize the benefits of cortisol control, resulting in improved comorbidities and better QoL for patients across all etiologies and severities of CS.

## Data Availability

Data sharing is not applicable to this article as no datasets were generated or analyzed during the current study.

## References

[1] Gadelha M, Gatto F, Wildemberg LE, Fleseriu M. Cushing's syndrome. Lancet. 2023;402(10418):2237‐2252. 37984386 10.1016/S0140-6736(23)01961-X

[2] Reincke M, Fleseriu M. Cushing syndrome: a review. JAMA. 2023;330(2):170‐181. 37432427 10.1001/jama.2023.11305

[3] Sharma ST, Nieman LK, Feelders RA. Cushing's syndrome: epidemiology and developments in disease management. Clin Epidemiol. 2015;7:281‐293. 25945066 10.2147/CLEP.S44336 PMC4407747

[4] Braun LT, Vogel F, Reincke M. Long-term morbidity and mortality in patients with Cushing's syndrome. J Neuroendocrinol. 2022;34(8):e13113. 35312199 10.1111/jne.13113

[5] Li D, Zhang CD, Saini J, et al Determinants of muscle function and health-related quality of life in patients with endogenous hypercortisolism: a cross-sectional study. Eur J Endocrinol. 2023;188(7):603‐612. 37327378 10.1093/ejendo/lvad069 PMC10376436

[6] Fleseriu M, Castinetti F. Updates on the role of adrenal steroidogenesis inhibitors in Cushing's syndrome: a focus on novel therapies. Pituitary. 2016;19(6):643‐653. 27600150 10.1007/s11102-016-0742-1 PMC5080363

[7] Hirsch D, Shimon I, Manisterski Y, et al Cushing's syndrome: comparison between Cushing's disease and adrenal Cushing's. Endocrine. 2018;62(3):712‐720. 30084101 10.1007/s12020-018-1709-y

[8] Wengander S, Trimpou P, Papakokkinou E, Ragnarsson O. The incidence of endogenous Cushing's syndrome in the modern era. Clin Endocrinol (Oxf). 2019;91(2):263‐270. 31094003 10.1111/cen.14014

[9] Pivonello R, Isidori AM, De Martino MC, Newell-Price J, Biller BM, Colao A. Complications of Cushing's syndrome: state of the art. Lancet Diabetes Endocrinol. 2016;4(7):611‐629. 27177728 10.1016/S2213-8587(16)00086-3

[10] Mondin A, Ceccato F, Voltan G, et al Complications and mortality of Cushing's disease: report on data collected over a 20-year period at a referral centre. Pituitary. 2023;26(5):551‐560. 37495935 10.1007/s11102-023-01343-2 PMC10539191

[11] Fleseriu M, Auchus R, Bancos I, et al Consensus on diagnosis and management of Cushing's disease: a guideline update. Lancet Diabetes Endocrinol. 2021;9(12):847‐875. 34687601 10.1016/S2213-8587(21)00235-7 PMC8743006

[12] Varlamov EV, Han AJ, Fleseriu M. Updates in adrenal steroidogenesis inhibitors for Cushing's syndrome—a practical guide. Best Pract Res Clin Endocrinol Metab. 2021;35(1):101490. 33707082 10.1016/j.beem.2021.101490

[13] Fleseriu M, Auchus RJ, Pivonello R, Salvatori R, Zacharieva S, Biller BMK. Levoketoconazole: a novel treatment for endogenous Cushing's syndrome. Expert Rev Endocrinol Metab. 2021;16(4):159‐174. 34380370 10.1080/17446651.2021.1945440

[14] Pivonello R, Zacharieva S, Elenkova A, et al Levoketoconazole in the treatment of patients with endogenous Cushing's syndrome: a double-blind, placebo-controlled, randomized withdrawal study (LOGICS). Pituitary. 2022;25(6):911‐926. 36085339 10.1007/s11102-022-01263-7 PMC9675660

[15] Recordati Rare Diseases . Osilodrostat prescribing information. 2023. Accessed November 2024. https://isturisa.com/wp-content/themes/isturisa-patient/dist/pdf/isturisa-prescribing-information.pdf

[16] Del Rivero J, Else T, Hallanger-Johnson J, et al A review of mitotane in the management of adrenocortical cancer. Oncologist. 2024;29(9):747‐760. 39037424 10.1093/oncolo/oyae084 PMC11379655

[17] Corcept Therapeutics Inc . Mifepristone prescribing information. 2019. Accessed November 2024. https://www.accessdata.fda.gov/drugsatfda_docs/label/2019/202107s008lbl.pdf

[18] De Martin M, Toja PM, Goulene K, et al No untoward effect of long-term ketoconazole administration on electrocardiographic QT interval in patients with Cushing's disease. Basic Clin Pharmacol Toxicol. 2016;118(4):279‐283. 26386326 10.1111/bcpt.12490

[19] HRA Pharma Rare Diseases . Ketoconazole HRA summary of product characteristics. 2021. Accessed November 2024. https://www.ema.europa.eu/en/documents/product-information/ketoconazole-hra-epar-product-information_en.pdf

[20] Recordati Rare Diseases . Pasireotide summary of product characteristics. 2024. Accessed November 2024. https://www.ema.europa.eu/en/documents/product-information/signifor-epar-product-information_en.pdf

[21] Recordati Rare Diseases . Osilodrostat summary of product characteristics. 2024. Accessed November 2024. https://www.ema.europa.eu/en/documents/product-information/isturisa-epar-product-information_en.pdf

[22] Latina Pharma SpA . Mitotane summary of product characteristics. 2024. Accessed November 2024. https://www.ema.europa.eu/en/documents/product-information/lysodren-epar-product-information_en.pdf

[23] Johanssen S, Allolio B. Mifepristone (RU 486) in Cushing's syndrome. Eur J Endocrinol. 2007;157(5):561‐569. 17984235 10.1530/EJE-07-0458

[24] Latina Pharma SpA . Mitotane prescribing information. 2024. Accessed November 2024. https://www.accessdata.fda.gov/drugsatfda_docs/label/2024/016885s033lbl.pdf

[25] Patra S, Dutta D, Nagendra L, Raizada N. Efficacy and safety of levoketoconazole in managing Cushing's syndrome: a systematic review. Indian J Endocrinol Metab. 2024;28(4):343‐349. 39371660 10.4103/ijem.ijem_477_23 PMC11451957

[26] Pivonello R, De Leo M, Cozzolino A, Colao A. The treatment of Cushing's disease. Endocr Rev. 2015;36(4):385‐486. 26067718 10.1210/er.2013-1048 PMC4523083

[27] Pivonello R, Simeoli C, Paola ND, Larocca A, Crescenzo EM, Colao A. Osilodrostat: a novel potent inhibitor of 11-beta-hydroxylase for the treatment of Cushing's syndrome. touchREV Endocrinol. 2024;20(1):43‐51. 38812665 10.17925/EE.2024.20.1.8 PMC11132648

[28] Recordati Rare Diseases . Pasireotide prescribing information. 2024. Accessed November 2024. https://www.accessdata.fda.gov/drugsatfda_docs/label/2024/200677s012lbl.pdf

[29] Recordati Rare Diseases . Pasireotide LAR prescribing information. 2024. Accessed November 2024. https://www.accessdata.fda.gov/drugsatfda_docs/label/2024/203255s011lbl.pdf

[30] Recordati Rare Diseases . Pasireotide LAR summary of product characteristics. 2024. Accessed November 2024. https://www.medicines.org.uk/emc/product/1932/smpc#gref

[31] Xeris Pharmaceuticals I . Levoketoconazole prescribing information. 2023. Accessed November 2024. https://www.accessdata.fda.gov/drugsatfda_docs/label/2023/214133s003lbl.pdf

[32] Yuen KCJ . Osilodrostat: a review of recent clinical studies and practical recommendations for its use in the treatment of cushing disease. Endocr Pract. 2021;27(9):956‐965. 34389514 10.1016/j.eprac.2021.06.012

[33] Esteve RD UK; Ireland Ltd. Metyrapone summary of product characteristics. 2023. Accessed November 2024. https://www.medicines.org.uk/emc/product/13897/smpc

[34] Hinojosa-Amaya JM, Cuevas-Ramos D, Fleseriu M. Medical management of Cushing's syndrome: current and emerging treatments. Drugs. 2019;79(9):935‐956. 31098899 10.1007/s40265-019-01128-7

[35] Varlamov EV, Langlois F, Vila G, Fleseriu M. Management of endocrine disease: cardiovascular risk assessment, thromboembolism, and infection prevention in Cushing's syndrome: a practical approach. Eur J Endocrinol. 2021;184(5):R207‐R224. 33539319 10.1530/EJE-20-1309

[36] Varlamov EV, Vila G, Fleseriu M. Perioperative management of a patient with Cushing disease. J Endocr Soc. 2022;6(3):bvac010. 35178493 10.1210/jendso/bvac010 PMC8845122

[37] Fleseriu M, Varlamov EV, Hinojosa-Amaya JM, Langlois F, Melmed S. An individualized approach to the management of Cushing disease. Nat Rev Endocrinol. 2023;19(10):581‐599. 37537306 10.1038/s41574-023-00868-7

[38] Creemers SG, Feelders RA, de Jong FH, et al Osilodrostat is a potential novel steroidogenesis inhibitor for the treatment of Cushing syndrome: an *in vitro* study. J Clin Endocrinol Metab. 2019;104(8):3437‐3449. 31127821 10.1210/jc.2019-00217

[39] Fleseriu M, Pivonello R, Young J, et al Osilodrostat, a potent oral 11β-hydroxylase inhibitor: 22-week, prospective, phase II study in Cushing's disease. Pituitary. 2016;19(2):138‐148. 26542280 10.1007/s11102-015-0692-z PMC4799251

[40] Bertagna X, Pivonello R, Fleseriu M, et al LCI699, a potent 11β-hydroxylase inhibitor, normalizes urinary cortisol in patients with Cushing's disease: results from a multicenter, proof-of-concept study. J Clin Endocrinol Metab. 2014;99(4):1375‐1383. 24423285 10.1210/jc.2013-2117

[41] Fleseriu M, Biller BMK, Bertherat J, et al Long-term efficacy and safety of osilodrostat in Cushing's disease: final results from a phase II study with an optional extension phase (LINC 2). Pituitary. 2022;25(6):959‐970. 36219274 10.1007/s11102-022-01280-6 PMC9675663

[42] Fleseriu M, Newell-Price J, Pivonello R, et al Long-term outcomes of osilodrostat in Cushing's disease: LINC 3 study extension. Eur J Endocrinol. 2022;187(4):531‐541. 35980235 10.1530/EJE-22-0317 PMC9513654

[43] Gadelha M, Bex M, Feelders RA, et al Randomized trial of osilodrostat for the treatment of cushing disease. J Clin Endocrinol Metab. 2022;107(7):e2882‐e2895. 35325149 10.1210/clinem/dgac178 PMC9202723

[44] Gadelha M, Snyder PJ, Witek P, et al Long-term efficacy and safety of osilodrostat in patients with Cushing's disease: results from the LINC 4 study extension. Front Endocrinol (Lausanne). 2023;14:1236465. 37680892 10.3389/fendo.2023.1236465 PMC10482037

[45] Pivonello R, Fleseriu M, Newell-Price J, et al Efficacy and safety of osilodrostat in patients with Cushing's disease (LINC 3): a multicentre phase III study with a double-blind, randomised withdrawal phase. Lancet Diabetes Endocrinol. 2020;8(9):748‐761. 32730798 10.1016/S2213-8587(20)30240-0

[46] Tanaka T, Satoh F, Ujihara M, et al A multicenter, phase 2 study to evaluate the efficacy and safety of osilodrostat, a new 11β-hydroxylase inhibitor, in Japanese patients with endogenous Cushing's syndrome other than Cushing's disease. Endocr J. 2020;67(8):841‐852. 32378529 10.1507/endocrj.EJ19-0617

[47] Amodru V, Brue T, Castinetti F. Synergistic cortisol suppression by ketoconazole-osilodrostat combination therapy. Endocrinol Diabetes Metab Case Rep. 2021;2021:21‐0071. 10.1530/EDM-21-0071 PMC8686175 34877930

[48] Auchus RJ, Fleseriu M, Huang W, et al Management, safety, and efficacy of osilodrostat treatment in US patients with non-pituitary Cushing's syndrome: results from the ILLUSTRATE study. J Endocr Soc. 2022;6(Supplement_1):A472‐A473.

[49] Bancos I, Geer EB, Castinetti F, et al A non-interventional, multinational, phase IV study to evaluate the long-term safety and efficacy of osilodrostat in patients with endogenous Cushing’s syndrome (LINC 6): 1-year real-world interim analysis. J Endocr Soc. 2024;8(Supplement_1):bvae163.171.

[50] Bessiène L, Bonnet F, Tenenbaum F, et al Rapid control of severe ectopic Cushing's syndrome by oral osilodrostat monotherapy. Eur J Endocrinol. 2021;184(5):L13‐L15. 33667191 10.1530/EJE-21-0147

[51] Bonnet-Serrano F, Poirier J, Vaczlavik A, et al Differences in the spectrum of steroidogenic enzyme inhibition between osilodrostat and metyrapone in ACTH-dependent Cushing syndrome patients. Eur J Endocrinol. 2022; 187(2):315‐322. 35699971 10.1530/EJE-22-0208

[52] Detomas M, Altieri B, Deutschbein T, Fassnacht M, Dischinger U. Metyrapone versus osilodrostat in the short-term therapy of endogenous Cushing's syndrome: results from a single center cohort study. Front Endocrinol (Lausanne). 2022;13:903545. 35769081 10.3389/fendo.2022.903545 PMC9235400

[53] Dormoy A, Haissaguerre M, Vitellius G, et al Efficacy and safety of osilodrostat in paraneoplastic cushing syndrome: a real-world multicenter study in France. J Clin Endocrinol Metab. 2023;108(6):1475‐1487. 36470583 10.1210/clinem/dgac691 PMC10188310

[54] Haissaguerre M, Puerto M, Nunes ML, Tabarin A. Efficacy and tolerance of osilodrostat in patients with severe Cushing's syndrome due to non-pituitary cancers. Eur J Endocrinol. 2020;183(4):L7‐L9. 32688343 10.1530/EJE-20-0557

[55] Hána V, Brutvan T, Krausová A, Kršek M, Hána V. Severe Cushing's syndrome from an ectopic adrenocorticotropic hormone-secreting neuroendocrine tumour treated by osilodrostat. Endocrinol Diabetes Metab Case Rep. 2023;2023(4):23‐0076. 10.1530/EDM-23-0076 PMC10620447 37855644

[56] Heleno CT, Hong SPD, Cho HG, Kim MJ, Park Y, Chae YK. Cushing's syndrome in adenocarcinoma of lung responding to osilodrostat. Case Rep Oncol. 2023;16(1):124‐128. 36876215 10.1159/000527824 PMC9978924

[57] Malik RB, Ben-Shlomo A. Adrenal Cushing's syndrome treated with preoperative osilodrostat and adrenalectomy. AACE Clin Case Rep. 2022;8(6):267‐270. 36447826 10.1016/j.aace.2022.10.001 PMC9701912

[58] Sawabe F, Hayafusa R, Kosugi R, Ariyasu H. A case of an ectopic ACTH-producing tumor with adrenal shrinkage during osilodrostat administration. JCEM Case Rep. 2024;2(2):luae008. 38283731 10.1210/jcemcr/luae008 PMC10821766

[59] Stasiak M, Witek P, Adamska-Fita E, Lewiński A. Response to osilodrostat therapy in adrenal Cushing's syndrome. Drug Healthc Patient Saf. 2024;16:35‐42. 38616817 10.2147/DHPS.S453105 PMC11011623

[60] Tabarin A, Haissaguerre M, Lassole H, et al Efficacy and tolerance of osilodrostat in patients with Cushing's syndrome due to adrenocortical carcinomas. Eur J Endocrinol. 2022;186(2):K1‐K4. 34905500 10.1530/EJE-21-1008

[61] Tabarin A, Bertherat J, Decoudier B, et al Safety and effectiveness of osilodrostat in patients with non-pituitary Cushing’s syndrome: results from the retrospective observational LINC 7 study. ENDO. 2024:SAT-671.

[62] Scheyer N, Bertherat J, Decoudier B, et al Effect of osilodrostat on cardiovascular and metabolic manifestations of hypercortisolism in patients with non-pituitary Cushing’s syndrome: findings from a retrospective observational study (LINC 7). J Endocr Soc. 2024;8(Supplement_1):bvae163.171.

[63] Fleseriu M, Molitch ME, Gross C, Schteingart DE, Vaughan TB 3rd, Biller BM. A new therapeutic approach in the medical treatment of Cushing's syndrome: glucocorticoid receptor blockade with mifepristone. Endocr Pract. 2013;19(2):313‐326. 23337135 10.4158/EP12149.RA

[64] Colao A, Block D, Gaztambide C, et al Managing hyperglycemia in patients with Cushing's disease treated with pasireotide: medical expert recommendations. Pituitary. 2014;17(2):180‐186. 23564338 10.1007/s11102-013-0483-3 PMC3942628

[65] Trementino L, Cardinaletti M, Concettoni C, Marcelli G, Boscaro M, Arnaldi G. Up-to 5-year efficacy of pasireotide in a patient with Cushing's disease and pre-existing diabetes: literature review and clinical practice considerations. Pituitary. 2015;18(3):359‐365. 24952218 10.1007/s11102-014-0582-9

[66] Genere N, Kaur RJ, Athimulam S, et al Interpretation of abnormal dexamethasone suppression test is enhanced with use of synchronous free cortisol assessment. J Clin Endocrinol Metab. 2022;107(3):e1221‐e1230. 34648626 10.1210/clinem/dgab724 PMC9006975

[67] Nieman LK, Biller BM, Findling JW, et al The diagnosis of Cushing's syndrome: an endocrine society clinical practice guideline. J Clin Endocrinol Metab. 2008;93(5):1526‐1540. 18334580 10.1210/jc.2008-0125 PMC2386281

[68] Sweeney AT, Hamidi O, Dogra P, et al Clinical review: the approach to the evaluation and management of bilateral adrenal masses. Endocr Pract. 2024;30:987‐1002. 39103149 10.1016/j.eprac.2024.06.015

[69] Bouys L, Violon F, Louiset E, Sibony M, Lefebvre H, Bertherat J. Bilateral adrenocortical nodular disease and Cushing's syndrome. J Clin Endocrinol Metab. 2024;109(10):2422‐2432. 38888184 10.1210/clinem/dgae419

[70] Iacobone M, Albiger N, Scaroni C, et al The role of unilateral adrenalectomy in ACTH-independent macronodular adrenal hyperplasia (AIMAH). World J Surg. 2008;32(5):882‐889. 18214589 10.1007/s00268-007-9408-5

[71] Sheikh-Ahmad M, Dickstein G, Matter I, et al Unilateral adrenalectomy for primary bilateral macronodular adrenal hyperplasia: analysis of 71 cases. Exp Clin Endocrinol Diabetes. 2020;128(12):827‐834. 31634962 10.1055/a-0998-7884

[72] Castinetti F, Guignat L, Giraud P, et al Ketoconazole in Cushing's disease: is it worth a try? J Clin Endocrinol Metab. 2014;99(5):1623‐1630. 24471573 10.1210/jc.2013-3628

[73] Fleseriu M, Pivonello R, Elenkova A, et al Efficacy and safety of levoketoconazole in the treatment of endogenous Cushing's syndrome (SONICS): a phase 3, multicentre, open-label, single-arm trial. Lancet Diabetes Endocrinol. 2019;7(11):855‐865. 31542384 10.1016/S2213-8587(19)30313-4

[74] Nieman LK, Boscaro M, Scaroni CM, et al Metyrapone treatment in endogenous Cushing’s syndrome: results at week 12 from PROMPT, a prospective international multicenter, open-label, phase III/IV study. J Endocr Soc. 2021;5(Supplement_1):A515.

[75] Daniel E, Aylwin S, Mustafa O, et al Effectiveness of metyrapone in treating Cushing's syndrome: a retrospective multicenter study in 195 patients. J Clin Endocrinol Metab. 2015;100(11):4146‐4154. 26353009 10.1210/jc.2015-2616 PMC5393433

[76] Shimatsu A, Biller BM, Fleseriu M, et al Osilodrostat treatment in patients with Cushing's disease of Asian or non-Asian origin: a pooled analysis of two phase III randomized trials (LINC 3 and LINC 4). Endocr J. 2024;71:1103‐1123. 39183039 10.1507/endocrj.EJ24-0153 PMC11778389

[77] Bancos I, Hahner S, Tomlinson J, Arlt W. Diagnosis and management of adrenal insufficiency. Lancet Diabetes Endocrinol. 2015;3(3):216‐226. 25098712 10.1016/S2213-8587(14)70142-1

[78] Castinetti F . Pharmacological treatment of Cushing's syndrome. Arch Med Res. 2023;54(8):102908. 37977919 10.1016/j.arcmed.2023.102908

[79] Pazderska A, Pearce SH. Adrenal insufficiency—recognition and management. Clin Med (Lond). 2017;17(3):258‐262. 28572228 10.7861/clinmedicine.17-3-258 PMC6297573

[80] Rushing GD, Britt RC, Collins JN, Cole FJ, Weireter LJ, Britt LD. Adrenal insufficiency in hemorrhagic shock. Am Surg. 2006;72(6):552‐554. 16808213

[81] Ferriere A, Salenave S, Puerto M, Young J, Tabarin A. Prolonged adrenal insufficiency following discontinuation of osilodrostat treatment for intense hypercortisolism. Eur J Endocrinol. 2024;190(1):L1‐L3. 38123490 10.1093/ejendo/lvad167

[82] Poirier J, Bonnet-Serrano F, Thomeret L, Bouys L, Bertherat J. Prolonged adrenocortical blockade following discontinuation of osilodrostat. Eur J Endocrinol. 2023;188(6):K29‐K32. 37300549 10.1093/ejendo/lvad060

[83] Barbot M, Ceccato F, Scaroni C. The pathophysiology and treatment of hypertension in patients with Cushing's syndrome. Front Endocrinol (Lausanne). 2019;10:321. 31164868 10.3389/fendo.2019.00321 PMC6536607

[84] Fleseriu M, Pivonello R, Newell-Price J, et al Continued improvements in hypertension and diabetes during long-term osilodrostat therapy in patients with Cushing’s disease: a pooled analysis from the phase III LINC 3 and LINC 4 studies. J Endocr Soc. 2024;8(Supplement_1):bvae163.1175.

[85] National Library of Medicine . Potassium test. 2023. Accessed October 2024. https://medlineplus.gov/ency/article/003484.htm

[86] Smushkin G, Phillips R, Chausse G. The elusive neuroendocrine tumor: finding the ectopic ACTH source 16 years after the diagnosis of cushing syndrome. JCEM Case Rep. 2023;1(1):luac035. 37908269 10.1210/jcemcr/luac035 PMC10578410

[87] Seastedt KP, Alyateem GA, Pittala K, et al Characterization of outcomes by surgical management of lung neuroendocrine tumors associated with Cushing syndrome. JAMA Netw Open. 2021;4(9):e2124739. 34586369 10.1001/jamanetworkopen.2021.24739 PMC8482056

[88] Young J, Haissaguerre M, Viera-Pinto O, Chabre O, Baudin E, Tabarin A. Management of endocrine disease: Cushing's syndrome due to ectopic ACTH secretion: an expert operational opinion. Eur J Endocrinol. 2020;182(4):R29‐R58. 31999619 10.1530/EJE-19-0877

[89] Amodru V, Ferriere A, Tabarin A, et al Cushing's syndrome in the elderly: data from the European registry on Cushing's syndrome. Eur J Endocrinol. 2023;188(4):395‐406. 36749009 10.1093/ejendo/lvad008

[90] Marques JVO, Boguszewski CL. Medical therapy in severe hypercortisolism. Best Pract Res Clin Endocrinol Metab. 2021;35(2):101487. 33518458 10.1016/j.beem.2021.101487

[91] Pence A, McGrath M, Lee SL, Raines DE. Pharmacological management of severe Cushing's syndrome: the role of etomidate. Ther Adv Endocrinol Metab. 2022;13:20420188211058583. 35186251 10.1177/20420188211058583 PMC8848075

[92] Wannachalee T, Turcu AF, Auchus RJ. Mifepristone in the treatment of the ectopic adrenocorticotropic hormone syndrome. Clin Endocrinol (Oxf). 2018;89(5):570‐576. 30019523 10.1111/cen.13818

[93] Corcuff JB, Young J, Masquefa-Giraud P, Chanson P, Baudin E, Tabarin A. Rapid control of severe neoplastic hypercortisolism with metyrapone and ketoconazole. Eur J Endocrinol. 2015;172(4):473‐481. 25624013 10.1530/EJE-14-0913

[94] Kamenický P, Droumaguet C, Salenave S, et al Mitotane, metyrapone, and ketoconazole combination therapy as an alternative to rescue adrenalectomy for severe ACTH-dependent Cushing's syndrome. J Clin Endocrinol Metab. 2011;96(9):2796‐2804. 21752886 10.1210/jc.2011-0536

[95] Castinetti F . Medical management of Cushing's disease: when and how? J Neuroendocrinol. 2022;34(8):e13120. 35348261 10.1111/jne.13120

[96] Newell-Price J, Fleseriu M, Pivonello R, et al Improved clinical outcomes during long-term osilodrostat treatment of Cushing's disease with LNSC and UFC normalization. J Endocr Soc. 2024:9;bvae201. 39610378 10.1210/jendso/bvae201 PMC11604051

[97] Bou Khalil R, Baudry C, Guignat L, et al Sequential hormonal changes in 21 patients with recurrent Cushing's disease after successful pituitary surgery. Eur J Endocrinol. 2011;165(5):729‐737. 21885674 10.1530/EJE-11-0424

[98] Torpy DJ, Mullen N, Ilias I, Nieman LK. Association of hypertension and hypokalemia with Cushing's syndrome caused by ectopic ACTH secretion: a series of 58 cases. Ann N Y Acad Sci. 2002;970(1):134‐144. 12381548 10.1111/j.1749-6632.2002.tb04419.x

[99] Fan L, Zhuang Y, Wang Y, et al Association of hypokalemia with cortisol and ACTH levels in Cushing's disease. Ann N Y Acad Sci. 2020;1463(1):60‐66. 31456238 10.1111/nyas.14205

[100] Fazio G, Vernuccio F, Grutta G, Re GL. Drugs to be avoided in patients with long QT syndrome: focus on the anaesthesiological management. World J Cardiol. 2013;5(4):87‐93. 23675554 10.4330/wjc.v5.i4.87 PMC3653016

[101] Fallahi B, Manafi-Farid R, Eftekhari M, et al Diagnostic efficiency of ^68^Ga-DOTATATE PET/CT as compared to ^99m^Tc-octreotide SPECT/CT and conventional morphologic modalities in neuroendocrine tumors. Asia Ocean J Nucl Med Biol. 2019;7(2):129‐140. 31380452 10.22038/AOJNMB.2019.39392.1263 PMC6661311

[102] Espinosa-de-Los-Monteros AL, Ramírez-Rentería C, Mercado M. Clinical heterogeneity of ectopic ACTH syndrome: a long-term follow-up study. Endocr Pract. 2020;26(12):1435‐1441. 33471735 10.4158/EP-2020-0368

[103] González Fernández L, Montenegro AMR, Torrecilla NB, et al Ectopic Cushing’s syndrome: clinical, diagnostic, treatment and follow-up outcomes of 12 cases of lung ectopic ACTH. Endocrinol Diabetes Metab Case Rep. 2023;2023:22‐0378.

[104] Theiler-Schwetz V, Prete A. Glucocorticoid withdrawal syndrome: what to expect and how to manage. Curr Opin Endocrinol Diabetes Obes. 2023;30(3):167‐174. 36876715 10.1097/MED.0000000000000804

[105] Zhang CD, Li D, Singh S, et al Glucocorticoid withdrawal syndrome following surgical remission of endogenous hypercortisolism: a longitudinal observational study. Eur J Endocrinol. 2023;188(7):592‐602. 37395115 10.1093/ejendo/lvad073 PMC10376560

[106] Fleseriu M, Biller BMK. Treatment of Cushing's syndrome with osilodrostat: practical applications of recent studies with case examples. Pituitary. 2022;25(6):795‐809. 36002784 10.1007/s11102-022-01268-2 PMC9401199

[107] He X, Findling JW, Auchus RJ. Glucocorticoid withdrawal syndrome following treatment of endogenous Cushing syndrome. Pituitary. 2022;25(3):393‐403. 35471718 10.1007/s11102-022-01218-y PMC9170649

[108] Turcu AF, Rege J, Auchus RJ, Rainey WE. 11-Oxygenated androgens in health and disease. Nat Rev Endocrinol. 2020;16(5):284‐296. 32203405 10.1038/s41574-020-0336-x PMC7881526

[109] Nowotny HF, Braun L, Reisch N. The landscape of androgens in Cushing's syndrome. Exp Clin Endocrinol Diabetes. 2024:132;670‐677. 38788777 10.1055/a-2333-1907

[110] Nowotny HF, Braun L, Vogel F, et al 11-Oxygenated C19 steroids are the predominant androgens responsible for hyperandrogenemia in Cushing's disease. Eur J Endocrinol. 2022;187(5):663‐673. 36074938 10.1530/EJE-22-0320 PMC9578081

[111] Pivonello R, Fleseriu M, Newell-Price J, et al Improvement in clinical features of hypercortisolism during osilodrostat treatment: findings from the phase III LINC 3 trial in Cushing's disease. J Endocrinol Invest. 2024;47(10):2437‐2448. 38696122 10.1007/s40618-024-02359-6 PMC11392997

[112] Nagendra L, Dutta D, Raizada N, Surana V, Selvan C, Bhattacharya S. Efficacy and safety of osilodrostat in managing Cushing's syndrome: a systematic review and meta-analysis. Indian J Endocrinol Metab. 2024;28(3):232‐238. 39086571 10.4103/ijem.ijem_260_23 PMC11288521

[113] Valentín-Goyco J, Liu J, Peng HM, Oommen J, Auchus RJ. Selectivity of osilodrostat as an inhibitor of human steroidogenic cytochromes P450. J Steroid Biochem Mol Biol. 2023;231:106316. 37098354 10.1016/j.jsbmb.2023.106316 PMC10757358

[114] Adkins KM, Lee JT, Bress AL, Spires SE, Lee CY, Ayoob AR. Classic Cushing's syndrome in a patient with adrenocortical carcinoma. Radiol Case Rep. 2013;8(3):826. 27330637 10.2484/rcr.v8i3.826 PMC4900127

[115] Kroiss M, Quinkler M, Lutz WK, Allolio B, Fassnacht M. Drug interactions with mitotane by induction of CYP3A4 metabolism in the clinical management of adrenocortical carcinoma. Clin Endocrinol (Oxf). 2011;75(5):585‐591. 21883349 10.1111/j.1365-2265.2011.04214.x

[116] Fassnacht M, Terzolo M, Allolio B, et al Combination chemotherapy in advanced adrenocortical carcinoma. N Engl J Med. 2012;366(23):2189‐2197. 22551107 10.1056/NEJMoa1200966

[117] Fassnacht M, Tsagarakis S, Terzolo M, et al European Society of Endocrinology clinical practice guidelines on the management of adrenal incidentalomas, in collaboration with the European network for the study of adrenal tumors. Eur J Endocrinol. 2023;189(1):G1‐42. 37318239 10.1093/ejendo/lvad066

[118] Saini J, Singh S, Ebbehoj A, et al Steroid profiling and circadian cortisol secretion in patients with mild autonomous cortisol secretion: a cross-sectional study. J Clin Endocrinol Metab. 2024:110;542‐553. 10.1210/clinem/dgae468 PMC11747667 38981002

[119] Debono M, Harrison RF, Chadarevian R, Gueroult C, Abitbol JL, Newell-Price J. Resetting the abnormal circadian cortisol rhythm in adrenal incidentaloma patients with mild autonomous cortisol secretion. J Clin Endocrinol Metab. 2017;102(9):3461‐3469. 28911138 10.1210/jc.2017-00823 PMC5587065

[120] Ortiz DI, Findling JW, Carroll TB, et al Cosyntropin stimulation testing on postoperative day 1 allows for selective glucocorticoid replacement therapy after adrenalectomy for hypercortisolism: results of a novel, multidisciplinary institutional protocol. Surgery. 2016;159(1):259‐265. 26422766 10.1016/j.surg.2015.05.034

[121] Fleseriu M, Biller BM, Findling JW, Molitch ME, Schteingart DE, Gross C. Mifepristone, a glucocorticoid receptor antagonist, produces clinical and metabolic benefits in patients with Cushing's syndrome. J Clin Endocrinol Metab. 2012;97(6):2039‐2049. 22466348 10.1210/jc.2011-3350

[122] Belokovskaya R, Ravikumar A, Arumugam D, et al Mifepristone treatment for mild autonomous cortisol secretion due to adrenal adenomas: a pilot study. Endocr Pract. 2019;25(8):846‐853. 31070948 10.4158/EP-2019-0047 PMC9125788

